# Digital Twins in Personalized Medicine: Bridging Innovation and Clinical Reality

**DOI:** 10.3390/jpm15110503

**Published:** 2025-10-22

**Authors:** Abigail Silva, Nuno Vale

**Affiliations:** 1 PerMed Research Group, RISE-Health, Faculty of Medicine, University of Porto, Alameda Professor Hernâni Monteiro, 4200-319 Porto, Portugal; abigailsilva@outlook.pt; 2 Laboratory of Personalized Medicine, Department of Community Medicine, Health Information and Decision (MEDCIDS), Faculty of Medicine, University of Porto, Rua Doutor Plácido da Costa, 4200-450 Porto, Portugal; 3 RISE-Health, Department of Community Medicine, Health Information and Decision (MEDCIDS), Faculty of Medicine, University of Porto, Rua Doutor Plácido da Costa, 4200-450 Porto, Portugal

**Keywords:** digital twins, personalized medicine, clinical decision support, precision health, translational medicine, healthcare innovation, regulatory and ethical challenges

## Abstract

Digital Twins (DTs) are poised to transform personalized medicine by enabling real-time, multiscale simulations of individual patients. By integrating genomics, imaging, wearable sensor data, and clinical records, DTs offer a powerful platform for predictive, adaptive, and patient-centered decision-making. Recent advances have highlighted their potential across a range of clinical domains, including cardiology, oncology, pharmacogenomics, and neurology. Yet, their routine application in clinical practice remains limited, underscoring a growing translational gap between digital innovation and healthcare delivery. In this review, we explore the scientific maturity and emerging clinical use cases of DTs, while critically analyzing the systemic, regulatory, ethical, and infrastructural barriers that hinder their widespread adoption. We outline a translational roadmap that emphasizes dynamic model validation, clinician co-development, equitable data representation, and regulatory harmonization. Uniquely, we reframe DTs as cognitive tools for clinical reasoning and decision support. We further clarify translational pathways through explicit evaluation and reporting recommendations. By positioning DTs within this practical framework, we outline how responsible, inclusive, and interdisciplinary implementation can establish them as foundational elements of 21st century precision medicine.

## 1. Introduction

Personalized medicine has emerged as one of the most transformative approaches in modern healthcare, marking a significant shift from population-based treatment strategies to patient-specific care. It aims to customize diagnostics, treatment choices, and follow-up plans to each person’s distinct biological, physiological, and genetic profile rather than using standardized interventions based on average responses [1,2,3,4,5].

The expanding range of high-resolution, high-throughput biomedical technologies, such as next-generation sequencing (NGS), proteomics, metabolomics, advanced medical imaging, and continuous monitoring devices, has fueled this evolution [3,6]. These tools have revealed an extraordinary degree of variability among individuals in how diseases manifest, progress, and respond to treatment. Yet, despite the vast amount of data now accessible from each patient, the true challenge lies in translating this complexity into clinically actionable decisions and doing so in real time [7].

This process of translation is still slow and not always smooth. Although data acquisition technologies have progressed rapidly, clinical systems have faced challenges in converting this information into customized care pathways. There is now a growing gap between what personalized medicine could do in theory and what healthcare systems can do at the bedside.

In this context, Digital Twins (DTs) have emerged as a promising approach to implementing personalized medicine in hospitals. In healthcare, DTs are personalized virtual models that show specific patients or their organs. This technology has been used in many fields for a long time, but its recent use in healthcare is especially interesting [8,9]. DTs can use real-time data to mimic the physiological and medical traits of each patient, such as their tissues, organs, and biokinetic parameters. This makes it easier to keep track of health and see how diseases are getting worse and how well treatments are working. Ultimately, digital twins hold promise for achieving truly personalized medicine by providing each patient with real-time, data-driven clinical decision support [4,8,10]. As a dynamic digital replica of the patient, the DT allows for modeling disease progression, anticipating treatment outcomes, and optimizing care pathways specific to the individual [11,12]. Beyond visualization, DTs serve as decision-support platforms that allow clinicians to test treatment strategies virtually, reducing risks, optimizing procedures, and enabling long-term monitoring [4,8].

**Figure 1:**
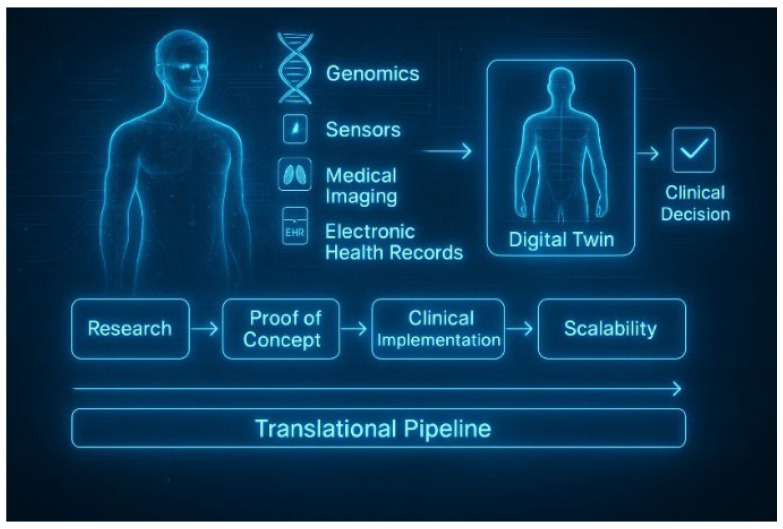
Overview of a Digital Twin in personalized medicine. Biomedical data sources, genomics, sensors, medical imaging, and electronic health records are integrated to create a virtual patient model that supports real-time clinical decisions. The lower section illustrates the translational pipeline from research to clinical scalability. Created with BioRender and improved with DALL·E (OpenAI), on 25 June 2025.

DTs create a direct link between the complexity of biomedicine and its clinical uses by combining various data sources, including wearable sensors, lab tests, medical imaging, artificial intelligence (AI) algorithms, and electronic health records. As a result, they are becoming increasingly recognized as one of the most promising technologies for achieving patient-centered, data-driven, and truly personalized care [5,10,13,14]. Figure 1 illustrates this process of integration, showing how multiple biomedical inputs converge into a DT to support real-time clinical decisions and outlining the translational pipeline toward clinical scalability.

Despite this potential, a significant obstacle still exists: the disconnect between innovation and real-world application. Despite an increasing amount of research demonstrating the theoretical and technical capabilities of digital technologies, including DTs, there is still a lack of actual integration of DTs in clinical settings. Few examples of current applications have made it into routine clinical practice; the majority are limited to pilot tests or experimental settings. It is still difficult to implement digital twins widely in routine clinical practice, despite the potential advantages. Although there are some promising pilot projects in fields like oncology and cardiology, expanding the use of DTs involves more than just technological developments [11,15,16,17]. Technical, legal, infrastructure, and ethical challenges are some of the causes of this predicament. To transform DTs from research models into useful clinical tools, it is imperative to comprehend this gap.

Strong digital infrastructure, comprehensive scientific validation, seamless interoperability, and an ethical framework that protects patient privacy and upholds confidence are all necessary. Nevertheless, incorporating Digital Twins into clinical environments signifies a crucial advancement toward a new chapter in personalized medicine, where every medical decision can be guided by a precise, contextual, and continuously updated simulation of each patient [18,19,20].

The objective of this review is to present and evaluate the clinical uses of digital twins, with an emphasis on how they contribute to individualized healthcare and the obstacles that prevent their widespread adoption. We aim to determine the achievements of Digital Twins as well as the current gaps between their potential and actual implementation by looking at cutting-edge research and fresh real-world examples.

This review critically examines how digital twins support personalized medicine by focusing on actual clinical use cases and patient-centered outcomes. It looks at real-world uses of these technologies in clinical settings, where their actual worth is evaluated, rather than just their theoretical potential. This conversation sets the stage for a thorough investigation of the Digital Human Twin (DHT), which serves as the theoretical foundation and impetus for the shift to personalized care. The successful integration of these tools into routine clinical procedures is crucial for long-term progress in this field, guaranteeing that breakthroughs result in notable improvements in patient health [21].

While existing reviews mainly map technical typologies and general use cases of Digital Twins, this manuscript makes a different contribution. First, it reframes DTs as cognitive tools that directly support clinical reasoning and decision workflows. Second, it distills the literature into clear translational criteria (validation strategies, reporting standards, regulatory considerations) that can be operationalized by researchers and clinicians. Together, these elements clarify what constitutes actionable clinical evidence for DTs, providing practical guidance that prior descriptive overviews have not offered.

## 2. Literature Search Strategy

This review was conducted as a structured narrative review. The literature was searched mainly in PubMed, Google Scholar, and ClinicalTrials.gov. Although no strict time filter was applied, particular attention was given to studies published in the last five years (2020–2025), reflecting the recent growth of clinical applications of digital twins.

The search was guided by keywords such as “digital twin clinics”, “digital twin cardiology”, “digital twin oncology”, “digital twin neurology”, “digital twin pharmacogenomics”, “digital twin rare diseases”, “digital twin clinical trials”, “digital twins in silico”, and “digital twins hospital”.

Inclusion criteria included studies on medical applications of digital twins, review articles on clinical or translational aspects, and original studies presenting case examples or validation methods. Exclusion criteria were purely technical or engineering-focused work lacking clinical relevance, non–peer-reviewed sources, conference abstracts without enough data, and duplicates.

An initial PubMed search using “digital twin clinics” retrieved around 630 records. After multiple rounds of screening and refinement based on the criteria above, only articles directly related to clinical applications, validation methods, or disease-specific implementations were kept. From this refined set, representative works from cardiology, oncology, neurology, pharmacogenomics, and rare diseases were chosen to reflect the current scope of progress.

This structured process ensured that the review is comprehensive while remaining focused on clinically relevant and translationally significant applications of digital twins.

## 3. State of the Art: Research Advances in Digital Twins

### 3.1. From Concept to Computational Reality

The creation of Digital Human Twins (DHT) represents a broader shift in medicine toward data-driven, individualized approaches. DHTs are dynamic, computational representations of specific patients that are constantly updated with real-time data, in contrast to conventional medical records or general population models [3,8,22,23,24,25]. They create accurate simulations that can predict health trends and treatment outcomes by combining a variety of sources, such as genomics, imaging, wearable sensors, clinical data, and lifestyle factors [4,9,11,26]. A new paradigm in biomedical modeling and decision-making is introduced by the transition from static electronic health records to dynamic, adaptive models. DHTs enable a significant change from reactive treatment to proactive, predictive, and individualized care by capturing the intricate interactions between physiological dynamics, anatomical structure, and molecular mechanisms [1,2,3,5,9]. Instead of just compiling data, these models build a computational environment where clinical hypotheses, interventions, and risks can be tested before real-world action is taken [11,27].

The capacity of DHTs to operate at different biological scales is one of their defining characteristics. They use high-resolution reconstructions of organs, such as the heart, brain, and lungs, using imaging methods like CT and MRI at the anatomical level [28,29]. Sophisticated biomechanical simulations, such as ventricular mechanics tailored to a patient for cardiac planning, are supported by this capability [29]. Furthermore, real-time sensor data and AI analytics frequently enhance the representation of physiological processes such as respiratory dynamics, cardiac conduction, and endocrine regulation through functional modeling [10,30,31].

DHTs use omics data, including transcriptomics, proteomics, and genomics, at the molecular level to guide targeted therapies, predict drug responses, and find genetic risk factors [27]. For example, cancer twins have been used in oncology to predict individual responses and model therapeutic pathways [27,31].

### 3.2. Model Typologies: Mechanistic, Data-Driven, and Hybrid Approaches

Based on the underlying modeling framework, DHTs can be categorized. Mechanistic models mimic organ function or systemic processes using well-established physiological and physical principles. Finite element models for cardiac simulations are one example of how these are frequently used in regulatory and surgical contexts and are usually interpretable [32,33]. AI-driven models, on the other hand, employ machine learning to identify trends and forecast outcomes from intricate, high-dimensional datasets [27,34]. Although they are effective at managing vast amounts of data, their interpretability may be restricted, which raises questions about clinical trust and transparency [10].

To address these limitations, hybrid models are emerging as a promising paradigm [10,34]. These allow explainable, adaptive, and scalable simulations by fusing the pattern-recognition powers of AI with the physiological coherence of mechanistic models. A mechanistic core preserves biological plausibility in such models, while machine learning can be used to stratify patient risk or personalize parameters [9].

### 3.3. Enabling Technologies: Infrastructure for Simulation and Personalization

The successful deployment of DHTs relies on a robust ecosystem of enabling technologies. The raw inputs come from data acquisition tools like wearable sensors, high-throughput sequencing platforms, and sophisticated imaging modalities [35,36]. Interoperability between health systems is made possible by data integration frameworks, such as FHIR (Fast Healthcare Interoperability Resources) and OMOP (Observational Medical Outcomes Partnership) standards, two different standards used in healthcare for data interoperability and standardization [2]. This data is converted into predictive models by simulation engines, which range from deep learning pipelines to finite element solvers [37,38,39,40]. Lastly, clinical interfaces, like decision-support systems (CDSS) and personalized dashboards, guarantee that the insights produced are understandable, actionable, and accessible to clinicians [41,42].

Cloud-based infrastructures and federated learning platforms are increasingly supporting the computational backbone of DHTs by enabling distributed model training while maintaining data privacy [43]. In order to scale DHTs beyond single-institution prototypes and make them tools appropriate for population-wide deployment, these infrastructures are essential.

Even though these technological developments are remarkable and getting more sophisticated, there is still little clinical application for them. These innovations are typically limited to early-stage pilot projects or controlled research settings. There is still a rare and uneven transition from laboratory proof-of-concept to routine clinical implementation, underscoring the ongoing and complex translational gap that divides potential from reality.

## 4. Clinical Applications of Digital Twins in Personalized Medicine

Over the past decade, DTs have moved from theoretical constructs into practical applications that are beginning to shape decision-making across various domains. Originally developed in engineering, aerospace, and architectural settings, where they support system monitoring, optimization, and predictive maintenance [44,45,46,47,48]. Several fields have shown concrete clinical use cases that highlight the potential of DTs to improve patient safety, increase treatment precision, and personalize care, even though their application in medicine remains largely in the experimental stage.

These applications now span a wide range of use cases, from diagnostics and treatment planning to procedural training and validation of implantable devices. They bring together geometric, physiological, and pharmacological calibration with retrospective clinical validation and, increasingly, real-time deployment. Though few qualify as full randomized trials, they collectively confirm the clinical viability of DTs, highlighting the importance of multimodal data integration, cross-disciplinary collaboration, and structured validation workflows that bridge model predictions with real-world evidence.

Among the medical specialties exploring DTs, cardiology has taken a leading role, thanks in part to the heart’s well-characterized physiology and the abundance of imaging and functional datasets available [29]. Patient-specific cardiac models are already being used to simulate electromechanical behavior, enabling clinicians to test various treatment strategies virtually before implementing them in the clinic. For instance, these models have been applied to optimize pacemaker lead placement in cardiac resynchronization therapy (CRT), tailoring interventions to individual anatomy and pathology [49,50].

**Figure 2:**
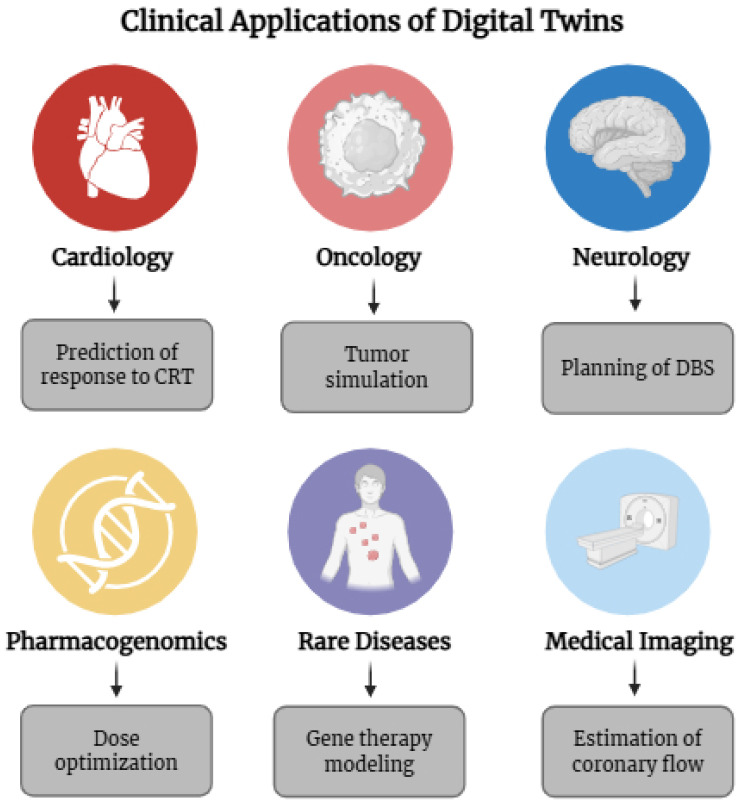
Clinical applications of Digital Twins (DTs) in personalized medicine by specialty, illustrating the main areas of activity: cardiology, oncology, neurology, pharmacogenomics, rare diseases, and medical imaging. Image created on BioRender on 21 June 2025.

DTs also show promise in supporting pharmacological decisions. In heart failure management, for example, real-time hemodynamic data combined with pharmacodynamic models enable clinicians to personalize titration of beta-blockers or diuretics. In one notable case, a finite element model of the left ventricle was used to predict the biomechanical impact of surgical remodeling, directly guiding a surgical plan. These examples point to a broader shift toward simulation-informed clinical decisions [51]. An overview of current clinical applications by specialty is summarized in Table 1. Figure 2 provides a visual summary of these applications across the main medical domains.

When it comes to CRT, multiscale and machine-learning-informed digital twins have been used to predict treatment response, with simulated values such as left ventricular contractility and QRS narrowing compared against outcomes captured post-implantation [52]. In a retrospective study of 260 patients, model predictions aligned with clinical results in over 70% of cases. Large retrospective cohorts showed predicted functional and echocardiographic responses in approximately 74% and 86% of CRT recipients, respectively. These outcomes suggest that, while not perfect, DTs are now sufficiently mature to inform complex treatment pathways [53].

In coronary artery disease, DTs have also entered clinical workflows. Platforms like HeartFlow, which estimate coronary flow reserve using CT angiography, have demonstrated clinical benefit across several trials. These studies showed a reduction in unnecessary invasive procedures, along with improvements in overall care efficiency and outcomes [54].

In oncology, where tumor heterogeneity complicates treatment planning, DTs are being used to simulate tumor growth and therapy response [4,27]. By combining imaging, histology, and genomic data, so-called “cancer avatars” allow clinicians to test different treatment strategies virtually before making decisions [55]. These models are particularly useful in radiation oncology and immunotherapy, where tailoring dose and sequencing can dramatically impact both toxicity and efficacy [56].

In oncology, cancer-specific DTs, or “cancer avatars,” have been created using a combination of tumor imaging, genomic profiles, and pharmacological parameters. These avatars simulate tumor evolution and therapy response across different treatment regimens, with retrospective comparisons showing convergence between simulated and actual tumor trajectories, particularly in breast cancer and melanoma [67,68]. Although their clinical use is still in its early stages, such models are now informing prospective trial design and individualized oncology workflows [59,67]. Additional pilot frameworks, such as TumorTwin, developed for glioma growth prediction, demonstrate the modular creation of cancer models ready for in silico experimentation [57]. Reviews published in 2024–2025 also confirm increasing adoption in diagnostic imaging and radiotherapy planning [27,58,65,66].

Gene therapy has also become a promising area for DT use, particularly in rare diseases. In spinal muscular atrophy (SMA), DTs have been used to simulate the biodistribution and immunogenicity of adeno-associated virus (AAV) vectors. DTs have modeled AAV vector distribution, expression levels, and immune responses in spinal muscular atrophy, with models prospectively validated against clinical trial data. In one case, predictions about transgene expression and immune response matched trial data closely [62,69]. Similarly, models that incorporate pharmacogenomic data (such as CYP450 polymorphisms) are now being used to optimize medication selection for conditions like depression and atrial fibrillation, reducing the risk of adverse effects [63].

Neurology and neurosurgery represent another frontier [63,64,65]. In Parkinson’s disease, DTs help guide electrode placement for deep brain stimulation (DBS) by modeling patient-specific circuit dynamics. In epilepsy, DTs derived from MRI, EEG, and tractography have helped simulate seizure spread and localize epileptogenic foci. A study showed that such a model successfully predicted the seizure origin, which was later confirmed and resected with positive clinical results. Similarly, neurovascular simulation models are being used to support procedural training with 4D-CTA-derived replicas [27,60,65,70].

AI-enhanced DTs are now also being tested for real-time clinical support. Surrogate deep-learning frameworks employing Neural ODEs achieve real-time whole-heart simulations, and transformer-based predictors within EHRs offer interpretable alerts for incident heart failure. These advances allow for continuous physiological modeling and earlier detection of clinical deterioration. On a systems level, broader initiatives are exploring how AI, XR (extended reality), and interoperability protocols can help scale DT deployment across healthcare systems. Reviews on Health Digital Twins have emphasized how combinations of AI, XR, and interoperability standards can support future clinical use in cardiology, with a focus on readiness for widespread adoption [59].

On a larger scale, researchers are beginning to explore how DTs can support population health and prevention. Conceptually, digital twins of entire populations could simulate disease evolution, evaluate public health strategies, and identify at-risk individuals early, though these approaches are still largely theoretical. Still, reviews in translational systems medicine and cyber-physical health highlight their potential [59].

Across all these areas, a few core enablers stand out: access to rich, multimodal clinical datasets; strong validation pipelines grounded in empirical outcomes; and close collaboration between clinicians, engineers, and data scientists. These initiatives form an essential foundation for future prospective trials using synthetic controls, adaptive methods, and regulatory-compliant validations. While few current efforts rise to the level of randomized trials, the groundwork being laid is strong and points toward robust clinical integration shortly.

Taken together, these case studies demonstrate that Digital Twins can be built, calibrated, and validated with sufficient fidelity to support real clinical decision-making. They provide blueprints not only for next-generation trials but also for designing validation strategies that are adaptive, data-rich, and consistent with regulatory expectations. As clinical questions become more complex and as computational tools continue to evolve, DTs are likely to become indispensable in the future of precision medicine.

## 5. The Translational Gap: From Conceptual Promise to Clinical Practice

The widespread incorporation of Digital Twins into standard clinical practice is still the exception rather than the rule, despite the mounting evidence in favor of DTs in personalized medicine. Although pilot projects and proof-of-concept studies have shown technical viability in a number of fields, such as neurology, cardiology, oncology, and pharmacogenomics, the shift from controlled to real-world settings has been sluggish, disjointed, and uneven [27,68,70,71].

This discrepancy highlights an important translational gap: the gap between the theoretical promise of DTs as reported in the scientific literature and their application as reliable, validated instruments that clinicians use daily. To advance the field, it is imperative to comprehend this gap.

### 5.1. Scientific Maturity vs. Clinical Readiness

There is no doubt about the maturity of DT science. The creation of complex models that can replicate integrated physiological responses, molecular pathways, and organ-level mechanics has exploded in the last ten years [3,9,29]. Nevertheless, rather than being used in clinical settings, these models are frequently tailored for academic performance.

Importantly, the criteria for clinical deployment (such as reproducibility, interoperability, explainability, and cost-effectiveness) are different from those for publishing a high-impact model (such as accuracy, novelty, and computational performance). Models produced as a result of this misalignment are technically stunning but functionally unrelated to actual clinical workflows.

Additionally, the majority of models have not been put to the test in clinical heterogeneity. Development datasets frequently do not include conditions like comorbidities, treatment polypharmacy, patient non-adherence, or socio-environmental determinants of health. As a result, when used in typical, noisy clinical settings, models that were trained in controlled environments might perform poorly [18].

This begs the crucial question: How can we move from models suitable for research to platforms that are ready for the clinic and can adjust to the complexity and unpredictability of actual human health?

### 5.2. Lack of Standardized Validation Frameworks

There is no widely recognized process for the validation and approval of digital twins, in contrast to pharmaceuticals or medical devices. Although initiatives like in silico clinical trials have been developed [37,38], they are still dispersed and do not agree on acceptable endpoints, generalizability thresholds, or reproducibility metrics.

For hybrid models, which blend mechanistic and artificial intelligence components, this is particularly problematic. Few evaluation protocols are prepared to handle the intersection of biological plausibility and statistical rigor, which are necessary for validating these systems [10,34].

Furthermore, current clinical trial approaches are not always appropriate for DTs. Although randomized controlled trials (RCTs) are still the gold standard for medical validation, their design may be ineffective or incompatible with evolving, data-driven, adaptive systems. Therefore, regulators need to develop and approve new trial architectures, such as digital cohort emulation, adaptive trials, and synthetic control arms [38,39].

DTs will find it difficult to obtain the clinical trust and regulatory status required for broad adoption in the absence of strong, open, and scalable validation pipelines.

### 5.3. Interoperability and Integration Barriers

DHTs are currently not supported by the technology architecture of the majority of healthcare facilities. Legacy systems that use antiquated or proprietary data formats, which restrict interoperability and scalability, are one of the primary obstacles. Clinical data is still dispersed across platforms and departments, resulting in silos that compromise cross-domain analysis and longitudinal modeling. Integration efforts are further complicated by disparate labeling or metadata standards among imaging repositories, sensor feeds, laboratory results, and electronic health records (EHRs) [72]. Complexity is increased by data latency, which hinders the real-time model updating necessary for dynamic, patient-specific simulations.

In addition to data fragmentation, there is a sizable computational infrastructure gap. Many hospitals lack the high-performance computing environments required to manage continuous data streams from wearables, bedside monitors, or home-based sensors, or to run high-fidelity simulations. Adoption is frequently hampered by worries about latency, cybersecurity, compliance, and cost, even in cases where cloud resources are technically available [73].

The difficulty of pre-processing and harmonizing data is a problem that is frequently disregarded. All inputs need to be cleaned, organized, normalized, and time-aligned for a DHT to work properly. This calls for specialized data engineering teams and clinical informatics knowledge, which are currently lacking in many healthcare facilities. In the end, these fundamental integration and infrastructure bottlenecks must be addressed if the promise of digital twins as real-time, adaptive clinical tools is to be fulfilled [74].

### 5.4. Explainability, Clinical Trust, and Decision Accountability

Explainability is a necessity, not a luxury, in clinical practice. Clinicians need to know how a particular prediction was made, which factors influenced the model’s output the most, and how uncertain the recommendation is. Even if the model’s predictive performance is high, clinicians may decide to disregard its recommendations if this information is lacking or ambiguous. In high-stakes decision-making situations, like neurosurgical planning, cardiovascular surgery, or oncology, where the human decision-maker bears the ultimate ethical and legal responsibility for results, this becomes especially problematic [42].

Furthermore, a fundamental tenet of contemporary healthcare is the collaborative decision-making process between physicians and patients. Digital twins’ outputs must be understandable to patients as well as medical professionals if they are to significantly influence clinical decisions. Current DT systems still struggle to achieve this dual-layer of explainability, which satisfies patient understanding and scientific rigor [75].

Additionally, accountability is becoming more and more ambiguous from a legal and ethical standpoint. It is unclear who is accountable if a clinician follows a DT’s advice and the patient suffers harm: the clinician, the software developer, or the medical facility. Building clinician trust and creating the institutional and legal frameworks required to support DT-guided care will be challenging without strong and open chains of decision responsibility [76].

### 5.5. Ethical and Regulatory Constraints

Digital twins present a number of ethical challenges that go far beyond data privacy issues. Consent is among the most urgent issues. New data sources are frequently incorporated into the model long after the initial consent procedure is finished because DHTs are dynamic and constantly updated. This calls into question whether consent needs to be renewed with every model iteration and whether patients are actually informed about how their data is being used over time [77].

Data sovereignty is another issue. It is frequently unclear who maintains control over the twins’ data when a patient moves institutions or countries. Predictive profiling may also reveal risks or disease predispositions that patients would rather not be aware of or that medical professionals are ill-equipped to appropriately discuss [78]. The potential for abuse also exists: could care be refused, particularly in systems that are cost-sensitive, if a Digital Twin indicates that a patient is unlikely to benefit from a specific treatment?

Technical guidelines and institutional review boards are insufficient to address these issues. They necessitate an international, multidisciplinary discussion and the creation of common ethical frameworks that strike a balance between patient safety and innovation [43,79]. Initiatives like the FDA’s Digital Health Center of Excellence and the EMA’s adaptive pathways show that regulatory bodies are starting to pay attention, but guidance is still disjointed, especially when it comes to long-term governance and cross-border implementation [78,80].

### 5.6. Health System Inequities and the Risk of Exclusion

Digital twins run the risk of becoming instruments of technological privilege unless inclusivity is purposefully incorporated into their creation. Because they have access to high-performance computing clusters, machine learning teams, and genomic biobanks, institutions in high-income nations are disproportionately positioned to gain from these advancements. In the meantime, DHT benefits may be routinely denied to patients in low- and middle-income nations, rural areas, or hospitals with inadequate resources [81].

Bias in training datasets makes this issue worse. When applied to diverse populations with varying genetic, socioeconomic, or environmental profiles, a Digital Twin’s performance may suffer if it was trained primarily on data from White, male, urban populations. This results in algorithmic inequity, which is a systematic mistake that may exacerbate already-existing disparities rather than just reflecting them [8,10,82].

The field needs to embrace equity as a fundamental design principle in order to reduce these risks. Technical standards for fairness and equitable performance must be established, diverse, representative training datasets must be created and curated, cloud-based Digital Twin infrastructures must be purchased, and regular bias audits must be required as part of model validation and clinical deployment. Equity cannot be treated as a downstream consideration; it must be structurally embedded into the Digital Twin ecosystem from the outset.

### 5.7. Organizational Culture and Clinical Workflow Disruption

Digital twin integration into healthcare systems is a major organizational shift rather than just a technical improvement. To facilitate the use of these technologies, hospitals and other clinical facilities need to rethink their internal workflows and structures [83]. This could entail developing completely new positions, like clinical AI leads, model interpreters, or coordinators for digital twins. To learn how to interact with model outputs, decipher predictions, and apply them to decisions that must be made quickly, clinicians will also require specialized training [84].

Furthermore, it is necessary to modify current clinical workflows to allow for simulation-informed decision-making without placing an excessive amount of strain on them. This is no easy task. Fear of professional redundancy, worries about an increased workload, or doubts about the accuracy of computational models are likely to be the main causes of resistance. These concerns are valid and must be addressed through thoughtful change management strategies.

To overcome this resistance, clinicians must be involved from the very beginning of the model’s design and implementation. Co-design guarantees usability, promotes a sense of ownership, and matches model outputs to actual clinical requirements. The only way to fully realize the transformative potential of digital twins is to integrate them into the daily culture of medicine, not just as tools but as partners [84].

### 5.8. Summary: Beyond the Technology

The translational gap is a complex systems challenge that involves culture, policy, ethics, and infrastructure, it is not just a technical problem. It captures the larger truth that healthcare innovation is not just about what we can create, but also about what we can apply, control, and maintain in practice [85,86].

Co-designed with clinicians, validated with patients, aligned with health systems, and transparently governed, the next stage of Digital Twin development must be translational by nature. Then and only then will DTs transform from digital promises into real clinical assets. These translational challenges span technical, regulatory, ethical, organizational, computational, and equity dimensions, as summarized in Table 2.

## 6. Clinical Trials and Validation Strategies for Digital Twins

Digital Twins must undergo rigorous clinical validation in addition to technical sophistication in order to move from experimental models to standard clinical tools. However, because traditional clinical trial frameworks were not made for dynamic, constantly updating, and data-driven technologies, DTs face a difficult conundrum in the current regulatory environment. New validation techniques are therefore needed, ones that combine the scalability and flexibility required by computational models with the robustness of traditional trials. The current initiatives, difficulties, and prospects in clinically validating DTs are examined in this section [87].

### 6.1. Why Are Conventional Trials Insufficient

The gold standard for evaluating clinical interventions is still randomized controlled trials, or RCTs. They frequently conflict with the patient-specific, adaptable nature of digital twins, though. Digital health technologies employ dynamic feedback loops, which are not reflected in static protocol designs. If customized simulations perform better than population-based protocols, standard control arms might be redundant or unethical. When clinicians work directly with model outputs, blinding becomes challenging. Because it can be difficult to separate the impact of a DT-guided decision from clinical judgment, outcome attribution is complicated. Full-scale RCTs have been conducted on relatively few DTs in practice, and those that have frequently been restricted to single centers or specialized applications [88].

### 6.2. Emerging Validation Approaches

Researchers are looking into different approaches that are better suited for validating digital health technologies in order to overcome these constraints. Computational models, frequently based on virtual cohorts, are used in in silico clinical trials to model the course of disease and the effects of treatment. They provide flexibility to refine trial designs before real-world deployment, the ability to simulate rare diseases or underrepresented populations, and quick hypothesis testing without endangering actual patients. For certain applications, especially in pharmacokinetics, device modeling, and rare diseases, an increasing number of regulatory agencies, such as the FDA and EMA, have started to accept in silico evidence as part of approval dossiers. When used in conjunction with conventional trials, in silico trials are particularly effective at validating and enhancing results [89,90].

Synthetic control arms mimic comparator populations using historical data or DT-generated simulations rather than by recruiting a conventional control group. In situations where the standard of care is already well established, this lessens the burden of recruitment and enhances ethical acceptability. By mimicking the disease trajectories of virtual patients who do not receive the experimental intervention, digital twins are essential in the creation of these artificial arms. When ongoing results change endpoints, eligibility requirements, or randomization ratios, DTs can help guide adaptive trial designs. The hybrid trial is a new idea where a real patient is mirrored by a digital twin and enrolled in a traditional clinical trial. The DT can be used to trigger alerts based on predicted risk, personalize treatment within the protocol, or simulate alternative outcomes. These tests enable a prospective comparison of model predictions and actual results, in addition to validating the DT [91].

### 6.3. Real-World Examples and Pilot Studies

Although extensive validation is still uncommon, a number of case studies demonstrate that DT-based validation is feasible. After being used to model reactions to cardiac resynchronization therapy, cardiac digital twins were subsequently verified using hemodynamic and echocardiographic data. By contrasting simulated tumor responses with actual results under chemotherapy or immunotherapy protocols, cancer avatars have been validated retrospectively. In gene therapy, the immune response and vector delivery simulations in spinal muscular atrophy were prospectively compared to the observed clinical results. These illustrations show that validation is feasible but necessitates multidisciplinary cooperation, data harmonization, and well-planned workflows [67,75,92].

### 6.4. Regulatory Perspectives and Frameworks in Development

The need for specific frameworks for digital health technologies is starting to be addressed by regulatory bodies. The FDA’s Precertification Program for Digital Health Software places a strong emphasis on practical performance and ongoing development. Early access and iterative evidence generation are promoted by the EMA’s Adaptive Pathways. Guidelines for the creation and evaluation of in silico models have been proposed by the Virtual Physiological Human initiative. However, the majority of DTs are in regulatory gray areas, making it difficult to determine whether they are research tools, clinical decision support, or software as a medical device (SaMD). This ambiguity can deter investment and cause translation delays [80,93]. In Europe, two recent regulations are particularly relevant for digital twins. The Medical Device Regulation (MDR, EU 2017/745) applies to software used in healthcare and requires proof of safety, clinical performance, and post-market monitoring [94]. As digital twins intended to support clinical decisions may therefore fall under the MDR as SaMD. Additionally, the new AI Act (2024) categorizes healthcare AI systems as high-risk and introduces requirements for transparency, risk management, and human oversight [95]. For digital twins that incorporate artificial intelligence, these regulations may create additional challenges, but they also provide clearer standards and can help build trust in clinical applications.

### 6.5. Toward a Validation Ecosystem for Digital Twins

It will take an integrated validation ecosystem with several essential components to achieve broad clinical adoption of DTs. Preclinical sandboxing describes settings for testing models under stress using patient data that has been anonymized. Digital sandboxes offer simulated clinical settings for comprehensive testing of the implementation of digital health technologies [96,97]. Federated validation networks allow multi-center collaborations to validate models across populations using privacy-preserving techniques like federated learning. Following clinical deployment, post-market surveillance is crucial for continuing to track DT performance, model drift, and outcome alignment. From inception to obsolescence, validation is an ongoing process that is integrated into a digital twin’s life cycle [98].

Despite these advances, significant challenges still exist. Since validation requires clean, labeled, longitudinal datasets, which are often difficult to obtain or share, data access and quality are essential. Because many real-world outcomes, like quality of life and symptom relief, are hard to model or record in DTs, outcome granularity presents obstacles. Furthermore, because new data is continuously added to digital health technologies, concerns about model stability and performance drift persist, making ongoing re-validation essential [99]. These issues underline that validation must be understood as a continuous, adaptive process rather than a one-time requirement.

## 7. Future Perspectives: From Proof-of-Concept to Clinical Standard

Digital Twins represent a cutting-edge approach to precision medicine, but their clinical integration continues to face substantial challenges that extend far beyond algorithmic performance. These challenges encompass scientific, infrastructural, ethical, legal, and organizational domains, and addressing them is essential to ensure that DTs evolve into sustainable and equitable tools for healthcare.

Importantly, regulatory harmonization will be needed to prevent duplication and expedite international implementation. A paradigm shift in clinical trials is also required, one that integrates co-validation, simulation, and adaptability as crucial elements of digital health assessment and this means moving beyond static evaluation frameworks toward flexible, context-aware approaches that regulators, clinicians, and patients can trust [99,100,101].

The validity and dependability of DT models in practical contexts are among the most urgent issues. Even though many DTs perform well in silico, their clinical robustness is frequently questionable when faced with the diversity of actual patients. Training datasets rarely capture the variables that are introduced in clinical settings, such as comorbidities, treatment variations, and adherence issues [102,103]. Concerns regarding generalizability and reproducibility are raised by the fact that many models are only validated within the organizations that created them. Furthermore, not many studies track long-term performance or take model drift into consideration as patients’ physiological conditions change. It is challenging to determine the clinical reliability of these systems in the absence of continuous performance monitoring and multi-institutional external validation.

Many DT models lack interpretability and transparency, which is an equally important obstacle. Clinicians may not be able to understand the reasoning behind certain predictions or treatment recommendations, especially in those that are based on deep learning or hybrid AI–AI-mechanistic architectures [10,34]. This opacity may restrict adoption in high-stakes situations like intensive care, cardiology, or oncology. Clinicians must defend their choices because they are held legally and ethically responsible for them [42,79].

These issues touch on serious moral dilemmas pertaining to consent and data privacy. Sensitive data, such as genetic, physiological, behavioral, and environmental streams, must be continuously ingested by digital twins. This makes dynamic consent more complicated, particularly as models develop and incorporate new kinds of data over time. Patients might not understand the uses of simulations or how their data is being processed. It becomes difficult to distinguish between surveillance and clinical monitoring. The technical framework for real-time consent management is still in its infancy, despite the stringent standards enforced by international data protection laws like the GDPR [20,43].

The possibility of escalating already-existing health disparities is another important concern. The best-positioned organizations to create and implement DTs are those with access to cutting-edge imaging, genomics, AI infrastructure, and qualified staff. On the other hand, environments with limited resources might not have the workforce, financial, or technical capacity to take part in or profit from these innovations. Simultaneously, training datasets frequently overrepresent the majority, wealthy, urban populations. By performing less accurately for underrepresented groups, this creates algorithmic bias, which exacerbates inequality rather than lessens it [8,10,82].

Integration into actual clinical workflows is still challenging, even in situations where regulatory approval has been obtained. The majority of hospital systems are dependent on non-interoperable platforms, fragmented data architectures, and outdated IT infrastructures. A DT needs to interface, often in real time, with imaging repositories, laboratory systems, wearable sensor platforms, and electronic health records to perform efficiently [2,35]. This degree of interoperability is rarely achieved. Furthermore, the current DT user interfaces are frequently not suited to the clinical setting; they may be too complicated, lack actionable summaries, or necessitate manual data entry. Even the most advanced DT runs the risk of being overlooked or underutilized in the absence of clinician-centered design [41,42].

Additional complications are introduced by legal responsibility and decision accountability. Who is responsible if harm results from a clinician following a DT’s advice, the doctor, the developer, or the organization? On the other hand, is it negligence if a clinician ignores a DT’s advice and a bad result occurs? To define the various functions and boundaries of algorithmic advice in patient care, these ambiguities necessitate precise legal definitions and institutional policies.

Last but not least, DTs question established organizational cultures in the medical field. Their introduction suggests new workflows, a learning curve for clinicians who might not be familiar with advanced modeling, and new roles (e.g., clinical model interpreters, digital twin managers). Adoption resistance may result from a lack of training time and resources, a fear of redundancy, or skepticism about automation. Therefore, to guarantee sustainable integration, change management techniques, such as education, co-design with clinicians, and incremental deployment, are essential.

In conclusion, obstacles that extend well beyond technological capacity influence the course of the broad adoption of digital twins. In addition to improved algorithms, addressing them calls for strong clinical validation, transparent regulations, ethical protections, workflow modifications, and a dedication to equity. The strategic directions and translational opportunities that can help close the gap between innovation and impact will be discussed in the section that follows [102,104].

## 8. Conclusion and Translational Outlook

A significant turning point in the development of personalized medicine has been reached with the introduction of Digital Twins in the healthcare industry. The modeling of patient-specific anatomy, physiology, and molecular dynamics has advanced significantly, as this review has shown. Promising clinical applications have been made possible by these advancements, ranging from pharmacogenomic therapy customization to tumor response prediction and cardiac intervention optimization. But the field is currently at a turning point, and its practical clinical integration has not yet caught up to the maturity of the underlying science [8,31].

The majority of DT systems are still limited to academic or experimental settings, despite their conceptual clarity and computational complexity. Inadequate technology is not the only cause of this translational lag. Instead, it results from a complex interaction of factors, including organizational resistance, regulatory ambiguity, insufficient interoperability, ethical uncertainties, and the absence of standardized validation pipelines. These restrictions run the risk of marginalizing DTs as impressive but unfeasible academic endeavors if they are not addressed [105].

However, this disparity also presents a chance. Clinical professionals, engineers, ethicists, regulators, health economists, and patients must work together as the field develops to create a strong translational foundation that puts clinical usability, safety, equity, and sustainability first. Several strategic directions can help bridge this translational divide [106].

The first step is to reframe validation as an iterative, dynamic, and context-aware process. The demands of real-world medicine, where patient states change constantly, cannot be satisfied by static models. Continuous performance monitoring integrated into learning health systems must replace strict, one-time evaluations in clinical trials for DTs. Hybrid trial designs provide scalable solutions for evidence generation without sacrificing ethical standards by combining parallel digital twin cohorts, adaptive methodologies, and synthetic controls [38].

Second, international harmonization and clarification of regulatory frameworks are required. A common classification system for DTs and modular approval pathways based on risk level, intended use, and autonomy should be the goals of organizations like the FDA, EMA, and WHO. Future regulatory expectations may include transparency requirements, audit trails, and model version control, particularly for autonomous or semi-autonomous systems [18,103].

Third, integration must be given top priority in technical development. Regardless of their predictive ability, DTs that are unable to integrate easily with clinical dashboards, imaging systems, and EHRs will continue to be underutilized. Co-developing clinical interfaces with frontline users and creating open, interoperable architectures should be prioritized [2,35,41].

Pathology offers a concrete proving ground for this integration imperative. Digital twins will recast pathology from a largely reactive pipeline into a predictive, continuously learning system that couples domain expertise with real-time evidence. Conceived as living software replicas of laboratory assets, processes, and even tissue or patient states, they allow teams to design and test workflows before deployment, mirror the behavior of individual instruments during routine use, and coordinate entire fleets of devices and tasks across the lab. In practice, this enables end-to-end situational awareness, from accessioning through grossing, embedding, staining, scanning, reporting, and archiving, where data streams feed models that anticipate bottlenecks, flag anomalies, and suggest optimal actions. Mislabeling can be driven toward near-zero through traceable routing; processing becomes more consistent as reagent dynamics and ergonomics are simulated and tuned; slide quality improves with predictive feedback; scanners balance loads and catch quality issues early; and AI-assisted “tissue/patient twins” accelerate triage, prognosis, and validation without displacing expert judgment. The enabling stack is deliberately pragmatic: LIS/EHR integration as the backbone; IoT telemetry for instruments and logistics; machine learning for anomaly detection and image quality assurance; and, where appropriate, cobots and RPA to automate repetitive handling while keeping humans in the loop for safety and nuance. Adoption should be phased and LIS-centric: first target high-impact slices such as traceability, stain quality, and scanner orchestration; then layer predictive maintenance and reagent analytics; then introduce AI-assisted diagnostic support; and finally extend the twin to archiving and compliance to close the lifecycle loop. Challenges are real but tractable: stage investments to deliver quick wins that self-fund expansion; embed training and co-design to drive workforce adoption; and enforce robust governance for privacy, security, and interoperability using open standards and, where needed, edge processing. Future work must strengthen longitudinal clinical and economic evidence and hone human-factors and ethics frameworks. If executed with this measured approach, digital-twin-enabled pathology can yield fewer errors, shorter turnaround, and more consistent quality, elevating pathologists to higher-value roles while making laboratories safer, smarter, and more patient-centered [107,108,109,110,111,112,113,114].

Fourth, as digital twins are used, the ethical framework governing them needs to change as well. DT design must incorporate mechanisms for equitable access strategies, clear delineation of data ownership, and dynamic, granular consent. To ensure that DTs do not exacerbate structural health inequities, training datasets must be diverse, bias must be explicitly monitored, and underserved populations must be included [8,43,82].

Fifth, clinical settings require cultural change and education. Digital ethics, algorithmic reasoning, and systems thinking ought to be incorporated into medical curricula. To facilitate the operationalization of DTs, healthcare organizations need to establish new positions and systems, such as AI governance boards, validation coordinators, and clinical model integrators. Even the most promising technologies will remain stuck in research silos in the absence of this ecosystemic shift [107].

Lastly, it is critical to ground DTs’ future in medicine’s overarching goal of enhancing patient outcomes, safety, and well-being. In the end, digital patient replicas must benefit actual people, not just theoretical algorithms. Their success should be evaluated based on significant, quantifiable, and equitable advancements in healthcare delivery rather than technical elegance.

Simply put, digital twins are no longer a sci-fi concept. They are turning into a clinical necessity. The scientific underpinnings are established. To turn innovation into impact and turn digital twins from experimental tools into commonplace assets in 21st-century medicine, a concerted effort is now required.

By providing customized, data-driven simulations of human physiology that can guide diagnosis, forecast results, and maximize treatment choices, digital twins are revolutionizing the field of personalized medicine. Multiscale model development, omics data integration, and proof-of-concept applications in cardiology, oncology, neurology, and pharmacogenomics have advanced significantly in recent years. The limited clinical use of DTs in spite of this quick scientific advancement, however, underscores the growing gap between innovation and execution [108].

In addition to critically examining the scientific, ethical, infrastructure, and regulatory obstacles that still impede adoption, this review looked at the state of the art in Digital Twin technologies and their most promising clinical applications. We contend that closing this gap will call for a fundamental rethinking of how digital models are verified, integrated, regulated, and governed in actual healthcare systems, in addition to technological advancement [52], and unlike prior descriptive reviews of Digital Twins that primarily catalog technological typologies or early applications, our synthesis provides two specific advances. First, it reframes DTs as cognitive tools for clinical reasoning and decision support, shifting attention from technical performance alone to their role in clinical epistemology and workflow integration. Second, it outlines translational criteria and reporting standards that clarify what constitutes actionable clinical evidence for DTs. This dual focus positions the present review as a bridge between conceptual potential and practical clinical adoption.

Future initiatives must place a high priority on dynamic validation techniques, clear regulations, ethical protections, health system interoperability, and equitable model design to fully utilize DTs. To guarantee usability, trust, and clinical relevance, co-development with patients and clinicians will be crucial. Ultimately, Digital Twins are not just tools for simulation; they are emerging instruments of clinical reasoning, capable of transforming care delivery if translated responsibly, inclusively, and at scale.

## Figures and Tables

**Table 1:** Clinical applications of Digital Twins (DTs) by medical specialty, including the type of model used and the current state of implementation.

Medical Specialty	Clinical Application	Implementation Status
Cardiology	Optimization of cardiac resynchronization therapy	Validated in studies [27,52,53,54]
Oncology	Tumor response simulation and personalized immunotherapy planning	Pilot/Experimental [27,53,55,56,57,58]
Neurology	Planning of deep brain stimulation in Parkinson’s disease	Experimental [59,60]
Pharmacogenomics	Dose adjustment based on genetic polymorphisms (CYP450)	Experimental [61,62,63]
Rare Diseases	Modeling of vector distribution in gene therapy	Pilot/Validated [63,64]
Medical Imaging	Coronary flow estimation via CT angiography (e.g., HeartFlow)	Clinical/Commercial [52,65,66,67]

**Table 2:** Main barriers to the clinical translation of Digital Twins (DTs), organized by type, with clinical impact and real-world examples.

Barrier Type	Description	Clinical Impact	Real-World Example
Technical	Lack of interoperability between hospital systems	Fragmented data, outdated models	EHRs are incompatible with sensors [72]
Regulatory	Absence of specific guidelines for DT approval	Delayed certification and institutional trust	No legal framework for hybrid models [37,38]
Validation	Lack of standardized validation frameworks	Lack of clinical trust, delays in adoption of the method	No agreed endpoints for in silico validation [10,34]
Ethical	Dynamic consent and continuous use of patient data	Risk of unauthorized or opaque use	Updates without renewed patient consent [43,77,78,79,80]
Trust/Explainability	Limited explainability and unclear decision accountability	Low clinician confidence, reluctant to use in practice	Opacity of AI-driven DT predictions in oncology/neurosurgery [42,75,76]
Organizational	Staff resistance and lack of training	Low adoption despite the availability of tools	Clinicians ignoring DT-generated alerts [84]
Computational	Insufficient infrastructure for real-time data processing	Inability to update the model dynamically	Hospitals lacking adequate servers or cloud resources [73]
Equity/Inclusion	Models trained on non-representative populations	Risk of algorithmic bias and errors in vulnerable populations	Underrepresentation of genetic and socioeconomic minorities [8,10,82]

## Data Availability

No new data were created or analyzed in this study. Data sharing is not applicable to this article.

## Abbreviations

The following abbreviations are used in this manuscript:

AAV	Adeno-Associated Viruses
AI	Artificial Intelligence
CDSS	Clinical Decision Support System
CRT	Cardiac Resynchronization Therapy
DBS	Deep Brain Stimulation
DHT	Digital Human Twin
DT	Digital Twin
EHR	Electronic Health Record
FHIR	Fast Healthcare Interoperability Resources
NGS	Next-Generation Sequencing
OMOP	Observational Medical Outcomes Partnership
RCT	Randomized Controlled Trial
SaMD	Software as a Medical Device
SMA	Spinal Muscular Atrophy
XR	Extended Reality
